# Translation and validation of the Korean confusion assessment method for the intensive care unit

**DOI:** 10.1186/1471-244X-11-94

**Published:** 2011-05-23

**Authors:** Eun Young Heo, Byoung-Jo Lee, Bong-Jin Hahm, Eun Hee Song, Han-A Lee, Chul-Gyu Yoo, Young Whan Kim, Sung Koo Han, Young-Soo Shim, Sang-Min Lee

**Affiliations:** 1 Seoul National University College of Medicine, 103 Daehangno, Jongno-gu, Seoul, 110-744, Republic of Korea

## Abstract

**Background:**

Delirium is a common problem and associated with poor outcomes in intensive care unit (ICU) patients. Diagnosis of delirium in ICU patients is limited and usually underdiagnosed by physicians. The Confusion Assessment Method for the Intensive Care Unit (CAM-ICU) is one of the most widely used screening methods for detection of ICU delirium. Our goal was to translate and validate the CAM-ICU for use in the Korean ICU setting.

**Methods:**

Translation of the CAM-ICU was done according to the guidelines suggested by the Translation and Cultural Adaptation Group. For validation and interrater reliability assessment of the Korean CAM-ICU, two nurses independently assessed delirium in ICU patients and the results were compared with the reference evaluation, which was done by a psychiatrist using the Diagnostic and Statistical Manual of Mental Disorders IV (DSM-IV).

**Results:**

Twenty-two patients were evaluated by two nurses and one psychiatrist expert independently. During the study period, we have continuously educated study nurses. Based on DSM-IV criteria, 16 out of 22 (72.7%) patients developed delirium. The sensitivities of the two nurses' evaluations using the Korean CAM-ICU were 89.80% for nurse 1 and 77.40% for nurse 2. Their specificities were 72.40% and 75.80% and their overall accuracy was 83.33% and 88.37% respectively. The Korean CAM-ICU was done with reasonable interrater reliability between nurse 1 and nurse 2 (κ = 0.81, *p *< 0.001).

**Conclusions:**

The Korean CAM-ICU showed good validity and could be incorporated into clinical practice in Korean ICUs.

**Trial registration:**

ISRCTN: ISRCTN50265663

## Background

Delirium is defined in the Diagnostic and Statistical Manual of Mental Disorders IV (DSM IV) as a disturbance of consciousness with inattention accompanied by a change in cognition or perceptual disturbance that develops over a short period and fluctuates over time [1]. Delirium is a common problem in patients in the intensive care unit (ICU) because of critical illness, medications, various procedures, and numerous risk factors [2]. Ely et al. reported that delirium occurred in between 81.7% and 87% of patients during their ICU stay [3-5]. Delirium itself is an independent predictor of mortality and longer hospital stay in ICU patients [5-7]. Therefore, the Society of Critical Care Medicine (SCCM) guidelines recommend routine assessment for the presence of delirium in ICU patients [8].

Despite the high prevalence and clinical importance of delirium in the ICU, detection of ICU delirium is limited, especially in mechanically ventilated patients, and delirium often goes undiagnosed by physicians [9,10]. The Confusion Assessment Method for the ICU (CAM-ICU) is a valid, reliable tool for detection of ICU delirium and is also used in nonverbal mechanically ventilated patients. It is simple, can be assessed by nonpsychiatrists with minimal training and takes only a few minutes [3,4,11]. Because of these benefits, the CAM-ICU has been translated into over a dozen languages but a Korean version of the CAM-ICU is not yet available. In this study, we attempted to translate and validate the CAM-ICU for practical use in the Korean ICU setting.

## Methods

### Patients

The Seoul National University Hospital institutional review board approved this study, and written informed consent was obtained from patients or their surrogates. The study population included both ventilated and nonventilated adult medical ICU patients admitted to the Seoul National University Hospital for longer than 24 hours. Exclusion criteria included patients who remained comatose throughout the investigation or were moribund, or who had a history of psychosis or neurologic disease that would confound the diagnosis of delirium. We also excluded the patient who had been already diagnosed as delirium before assessment and been prescribed antipsychotics. The study was conducted in March 2009.

### Translation and Back-translation

After permission from Ely et al., translation of the instrument was carried out according to the guidelines suggested by the Translation and Cultural Adaptation group [12,13]. The CAM-ICU was translated into Korean by the authors--doctors of pulmonology, psychiatrists and Masters students majoring in English. Each carried out their translation independently and then these were discussed. The final Korean version was given to a professional translator for back-translation to English without any information about the original version. The back-translated version was sent to Ely et al. for approval and acceptance of the Korean version.

### Validation of Delirium Assessment and Interrater Reliability

One research nurse and another experienced nurse specializing in intensive care independently conducted delirium assessment in the enrolled patients using the Korean version of the CAM-ICU (Korean CAM-ICU). For reference standard evaluation, an experienced psychiatrist (L-BJ) specializing in consultation psychiatrics independently assessed the delirium using the DSM-IV criteria. All assessments were done between three and seven o'clock in the afternoon to avoid any bias arising from changes in patients' condition.

To validate the Korean version, we compared the Korean CAM-ICU users to the psychiatrist ratings of delirium using the DSM-IV criteria as the reference standard. For interrater reliability, we compared the Korean CAM-ICU ratings between the two nurses by κ coefficient.

During the study period, we had times to discuss about the rating results with two nurses and the psychiatrist. In addition, we continuously educated study nurses regarding any mistakes or misconceptions. However, any rating results which had been already made were not changed after these processes.

## Results

### Patient Characteristics

During the 1-month study period, assessments were conducted for 16 days and 22 patients were analyzed. On each assessment day, we screened all patients admitted to medical ICU and enrolled patients who were satisfied with inclusion criteria.

The baseline characteristics of the patients are summarized in Table 1. Most patients (63.6%) were male and the median age was 68 years (range: 19-87). The most common cause of ICU admission (40.9%) was acute respiratory distress syndrome (ARDS). The median APACHE (Acute Physiology and Chronic Health Evaluation) II at admission was 25.5 (range: 9-39). Based on DSM-IV criteria, 16 out of 22 (72.7%) enrolled patients developed delirium at least once during the evaluation period.

**Table 1:** Baseline characteristics of the study population

Characteristics	Frequency (Total N = 22)
**Male(%)**	14 (63.6)
**Age, median(range)**	68 (19-87)
**Cause of ICU admission (%)**	
**ARDS**	9 (40.9)
**Sepsis**	3 (13.6)
**Cardiac**	3 (13.6)
**Airway disease**	4 (18.2)
**Others**	3 (13.6)
**APACHEII, median(range)**	25.5 (9-39)
**Delirium using DSM-IV (%)**	16 (72.7)

### Interrater reliability and validity of the Korean CAM-ICU

Ninety-six paired comparisons were conducted in 22 patients. Every enrolled patient was assessed more than once. Average number of assessments per patients was 4.7 and three patients were assessed more than 10 times.

For interrater reliability, nurse2 recorded feature 1 as positive in all ratings, so we were unable to determine the kappa value of feature 1. There were disagreements about the feature 1 in 22 out of 96 cases (23%) between two nurses. The kappa values of the other features were 0.91, 0.60 and 0.64 respectively (Table2). The Korean CAM-ICU was done with reasonable interrater reliability, considering all 4 items, between nurse 1 and nurse 2 (κ = 0.81, *p *< 0.001).

**Table 2:** Interrater reliability of each part of the Korean CAM-ICU

Component of CAM-ICU		N = 96*	
		*Kappa*	*p-value*
**Feature I**	Acute onset or fluctuating course	.	.
**Feature II**	Inattention	0.91	<0.001
**Feature III**	Disorganized thinking	0.60	<0.001
**Feature IV**	Altered level of consciousness	0.64	<0.001

^* ^Interrater reliability measures across 96 paired comparison showed **kappa of 0.81 (p-value <0.001)**.

### Validity of the Korean CAM-ICU

Patients who were admitted to the medical ICU more than 24 hours previously were evaluated. During the study period, 74 paired evaluations were done between nurse 1 and the psychiatric expert and 86 paired evaluations were done between nurse 2 and the psychiatric expert. The sensitivities of the two nurses' evaluations using the Korean CAM-ICU compared with the reference standard were 89.80% for nurse 1 and 77.40% for nurse 2. Their specificities were 72.40% and 75.80% and their overall accuracies were 83.33% and 88.37% respectively (Table 3).

**Table 3:** Validity of the Korean CAM-ICU

Rater	No. of paired observations	Sensitivity,%	Specificity,%	Overall accuracy,%
Nurse_1*	78	89.80	72.40	83.33
Nurse_2*	86	77.40	75.80	88.37

Korean CAM-ICU comparisons were made to reference standard evaluations by psychiatric expert using Diagnostic and Statistical Manual of Mental Disorders, Fourth Edition, criteria.

## Discussion

The sensitivity and specificity of the Korean CAM-ICU was 89.8% and 77.4%, respectively, for nurse 1 and 72.4% and 75.8% for nurse 2. Overall agreement (κ) between nurse 1 and nurse 2 was 0.81 (*p *< 0.001). Compared with the original validation study of the CAM-ICU (sensitivity = 100%, 93%, specificity = 98%, 100%) [3], the Korean CAM-ICU showed a little lower sensitivity, specificity and kappa value. Ely et al., who originally devised the CAM-ICU, explained that the assessors also interviewed patients' family members to estimate their baseline mental status [3,4]. In practice, interviews with patients' families are not easy and our study nurses could not perform such family interviews. Feature 1 is the one of 2 essential components for diagnose delirium using the CAM-ICU. Therefore, knowing the patient's baseline mental status is very important thing. In this study, we couldn't get direct information of patients' baseline mental status from patients' family and just presumed from previous medical records. That might be one of limitations of this study.

Ely et al. conducted a study to determine the feasibility of implementing the CAM-ICU [14]. Overall agreement (κ) between two different hospitals (one the hospital where the original validation study of the CAM-ICU was performed) was initially very low (κ = 0.2, 0.03). However, this was much improved (κ = 0.92, 0.75, respectively) through an education period.

We have several limitations in this study. First, we don't have exact number and detailed clinical information of excluded patients even though we screened all patients admitted to medical ICU. This may have an effect on the representativeness of enrolled patients and the results of this study. Second, most patients had been assessed several times and the data might be correlated and independent. Third, the time between the assessments was various. While some patients had been evaluated for 3 to 4 consecutive days, other patients were evaluated at admission and then just before leaving the ICU due to their medical conditions.

## Conclusions

The Korean version of the CAM-ICU showed good validity and could be incorporated into clinical practice in Korean ICUs. However, we should remember that sufficient education and a feedback process are needed during the introductory period. We hope that the Korean CAM-ICU will help clinicians detect delirium in the ICU and eventually improve the outcome of patients in the ICU through reducing the incidence of delirium and its potential complications.

## List of abbreviations

ICU: Intensive Care Unit; CAM-ICU: The Confusion Assessment Method for the Intensive Care Unit; DSM-IV: The Diagnostic and Statistical Manual of Mental Disorders IV; APACHE II: Acute Physiology And Chronic Health Evaluation II; ARDS: Acute Respiratory Distress Syndrome.

## Competing interests

The authors declare that they have no competing interests.

## Authors' contributions

HEY: analysis of data and manuscript preparation. LBJ and HBJ: assessment of delirium and analysis of data. SHE and LHA: important contribution to acquisition of data. YCG, KYW HSK and SYS: interpretation of data and manuscript review. LSM: study design, interpretation of data and manuscript preparation.

All authors read and approved the final manuscript.

## Pre-publication history

The pre-publication history for this paper can be accessed here:

http://www.biomedcentral.com/1471-244X/11/94/prepub
